# Correlation between diagnostic nasal endoscopy and computed tomography scans of patients with chronic rhinosinusitis: A cross-sectional study

**DOI:** 10.1097/MD.0000000000045748

**Published:** 2025-11-07

**Authors:** Thanh Nam Quan, Duc Thuan Nghiem, Tram Anh Do

**Affiliations:** a Department of Otorhinolaryngology, 103 Military Hospital, Vietnam Military Medical University, Hanoi, Vietnam.

**Keywords:** chronic rhinosinusitis, computed tomography scans of sinuses, diagnostic nasal endoscopy

## Abstract

The diagnosis of chronic rhinosinusitis (CRS) is usually based on a combination of symptoms, diagnostic nasal endoscopy (DNE), and computed tomography scan (CT scan). This study evaluates the correlation between DNE and CT scans in patients with CRS. This cross-sectional study included 52 patients diagnosed with CRS based on EPOS 2020 guidelines. All patients underwent DNE followed by CT scans and all the findings were assessed by the Lund-Kennedy and Lund-Mackay scoring system. Participants had an average age of 51.57 ± 15.58 years, with a male-to-female ratio of 2:1. Common symptoms included nasal discharge (96.2%), nasal congestion (86.5%), and sneezing (67.3%). The average Lund-Kennedy score was 3.83 ± 1.37 and the average Lund-Mackay score was 4.3 ± 2.4. DNE demonstrated high sensitivity (95.65%) and specificity (83.33%) compared to CT, with a positive predictive value of 97.78% and negative predictive value of 71.43%. DNE has been shown to correlate effectively with CT scans as a valuable diagnostic tool in the management of CRS. DNE is a valuable first-line diagnostic tool in CRS and can reduce the need for CT in appropriate cases.

## 1. Introduction

Chronic rhinosinusitis (CRS) is a common disease in the otorhinolaryngology field that results in significant impairment of quality of life. CRS is defined as an inflammatory disease of the mucosa of the nose and paranasal sinuses that lasts at least 3 consecutive months. The primary pathogenesis of CRS involves obstruction of the ostiomeatal complex, which is clinically manifested by the main symptoms of nasal blockage, obstruction, or congestion; anterior or posterior nasal drainage; facial pain or pressure; and hyposmia with objective findings on imaging or nasal endoscopy.^[1]^

Diagnostic nasal endoscopy (DNE) and computed tomography (CT) scans are crucial diagnostic tools for nasal and paranasal sinus diseases. DNE provides an objective assessment of the mucosa and directly sees changes in structures such as nasal cavity deformities, nasal polyps, and secretions. This technique helps in the early categorization of patients for further evaluation by imaging in the diagnosis, treatment decisions, and prognosis.^[2]^ However, the limitation of this procedure is the difficulty in diagnosing localized conditions within the infundibulum, frontal recess, and maxillary sinus ostium.^[3]^ In contrast, the advent of CT scanning has substantially enhanced diagnostic capabilities for CRS, and it is widely accepted as the gold standard when considering functional endoscopic sinus surgery, evaluating suspected complications of sinusitis and neoplasms of the nose and paranasal sinuses.^[4–6]^ This is particularly relevant in cases where endoscopic evaluation does not reveal disease. Nevertheless, drawbacks of CT include radiation exposure, high costs, and elevated rates of false positives.^[7,8]^

Many clinicians regard CT scans as the gold standard in the diagnosis of CRS but it is not widely available in resource-poor countries. Consequently, CT is typically indicated after failed medical treatment, when surgical intervention is planned, or in the presence of complications. Multiple comparative studies have assessed the diagnostic utility of CT and DNE in CRS, demonstrating that both modalities are effective for the diagnosis and management of this condition. However, the role of endoscopy and whether it can replace CT scans in the effective assessment of CRS remains a topic of discussion. Therefore, the present study aims to evaluate the correlation between DNE and CT scanning in diagnosing patients with CRS.

## 2. Materials and methods

### 2.1. Subjects

This study was approved by the Institutional Review Board of 103 Military Hospital, Hanoi, Vietnam. Informed consent was obtained from all participants prior to enrollment. Fifty-two patients diagnosed with CRS were recruited in the Department of Otorhinolaryngology at 103 Military Hospital from June 2023 to June 2024. Patients were clinically diagnosed with CRS in accordance with the European Position Paper on Rhinosinusitis and Nasal Polyps (2020) standards.^[9]^ All of them underwent diagnostic procedures, including DNE and CT scans. Complete medical records were maintained, and full informed consent was obtained from all participants. Exclusion criteria included patients without a diagnosis of CRS and those unable to undergo the aforementioned diagnostic procedures.

### 2.2. Outcome measures

All the patients were diagnosed with CRS (with or without nasal polyps) which is defined as the presence of 2 or more symptoms, one of which must be either nasal blockage, obstruction, congestion, or nasal discharge (anterior/posterior nasal drip): along with possible facial pain or pressure and/or reduced or lost sense of smell. Symptoms must have persisted for more than 12 weeks. The procedure of DNE was performed prior to the CT scans to eliminate any observer bias. Rigid nasal endoscopy using 0° and 30° Medtronic 4 mm diameter was conducted on all subjects. The endoscopic findings were interpreted using the Lund-Kennedy grading system to assess the following parameters in Table 1.^[10]^ Endoscopy was categorized as normal or “negative” (Endo-) if Lund-Kennedy score was 0; any other score indicated abnormal findings and was classified as “positive” (Endo+).

**Table 1:** Lund-Kennedy grading system of endoscopic assessment.

Endoscopic assessment	Characteristics	Scores
Nasal mucosal edema	Absent	0
	Minimal	1
	Presence of secretion	2
Presence of secretion	Absent	0
	Thin and clear discharge	1
	Thick and purulent	2
Presence of polyps	Absent	0
	Restricted to the middle meatus	1
	Present in the nasal cavity but not obstructing the airway	2

Patients underwent CT scans using a 64-slice CT machine, with 1.5 mm thin cuts obtained in coronal, sagittal, and axial planes, evaluated under both bone-window and soft-tissue window settings. The CT scans were assessed using the Lund-Mackay system, as outlined in Table 2.^[11]^ This scoring system ranges from 0 (complete lucency of all sinuses) to 24 (complete opacity of all sinuses). A score of 0 for the sinuses and ostiomeatal complex was classified as normal or “negative” (CT‐), while scores above 0 were categorized as abnormal or “positive” (CT+).

**Table 2:** Lund-Mackay system of CT scans assessment.

CT scans assessment	Characteristics	Scores
The right or left sinuses respectively, including maxillary sinus, anterior ethmoid sinuses, posterior ethmoid sinuses, sphenoid sinus, frontal sinus	Complete lucency	0
	Partial lucency	1
	Complete opacity	2
The ostiomeatal complex	Not obstructed	0
	Obstructed	2

### 2.3. Statistical analyses

Data were analyzed using IBM SPSS Statistics for Windows, version 22.0 (IBM Corp., Armonk). For statistical analysis, data are presented as the mean ± standard deviation. Values of *P* < .05 were considered statistically significant. Sensitivity, specificity, positive predictive value (PPV), and negative predictive value (NPV) of nasal endoscopy about the CT scan findings were determined.

## 3. Results

**Figure 1.**
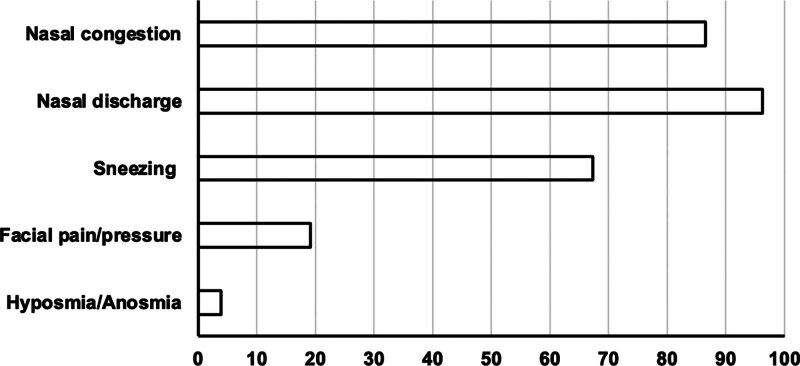
Prevalence of symptoms of patients.

The patients were predominately male, with 35 males (67.1%) and 17 females (32.7%). The mean age of the CRS patients was 51.57 ± 15.58 years (range, 22–83 years). The prevalence of symptoms in patients with CRS is illustrated in Figure 1. In this study, the majority of the patients had nasal discharge (96.2%) and nasal congestion (86.5%) as the chief complaints, while sneezing was noted in 67.3% of cases. Facial pain or pressure and hyposmia/anosmia were reported less frequently, at 19.2% and 3.9%, respectively.

The diagnostic findings of DNE and CT scans are detailed in Table 3. Endoscopic examination revealed that 70.6% of patients had nasal mucosal edema, with bilateral edema observed in 61.0% of cases. Furthermore, 34.6% of patients presented with nasal polyps, and 61.6% exhibited nasal discharge. The average Lund-Kennedy score was 3.83 ± 1.37. Lesions in the ostiomeatal complex were observed in 67.3% of patients, while lesions in the maxillary sinus and ethmoid sinuses were noted in 61.6% and 57.7%, respectively. The average Lund-Mackay score was 4.3 ± 2.4.

**Table 3:** Nasal endoscopy and CT scans findings.

Sinuses	One side	Both sides	Total
	n (%)	n (%)	N (%)
Nasal endoscopy findings			
Nasal mucosal edema	5 (9.6)	47 (61.0)	52 (70.6)
Presence of secretion	4 (7.7)	25 (48.1)	32 (61.6)
Presence of polyps	6 (11.5)	12 (23.1)	18 (34.6)
Lund-Kennedy score (mean ± SD)	3.83 ± 1.37		
CT scans findings			
Maxillary sinus	7 (13.5)	25 (48.1)	32 (61.6)
Frontal sinus	1 (1.9)	3 (5.8)	4 (7.7)
Ethmoid sinuses	9 (17.3)	21 (40.4)	30 (57.7)
Sphenoid sinus	5 (9.6)	3 (5.8)	8 (15.4)
The ostiomeatal complex	10 (19.2)	25 (48.1)	35 (67.3)
Lund-Mackay score (mean ± SD)	4.3 ± 2.4		

The overall correlation between nasal endoscopy and CT scan findings is shown in Table 4. Most of the positive endoscopy patients of CRS were CT positive with 44 cases, while 1 case was negative with CT. Out of the 46 positive CT cases, 2 were not detected by endoscopy and 5 cases were negative on both modalities. There was a significant association between endoscopic and CT findings in the diagnosis of CRS. The sensitivity of endoscopy was 95.65% and the specificity was 83.33%. The PPV was 97.78% and the NPV was 71.43%. The accuracy of the test was 95% (Table 5).

**Table 4:** The overall correlation between nasal endoscopy and CT scan findings.

	CT+	CT‐	Total	*P*-value
Endo+	44	1	45	.00001
Endo‐	2	5	7	
Total	46	6	52	

**Table 5:** Sensitivity and specificity.

Parameters	%
Sensitivity	95.65
Specificity	83.33
Positive predictive value	97.78
Negative predictive value	71.43
Accuracy	95

## 4. Discussion

Chronic rhinosinusitis is a common disease diagnosed based on a combination of symptoms, nasal endoscopy, and/or CT scan imaging. While CT scans are considered the gold standard for diagnosis and surgical planning for CRS, endoscopic findings provide the direct observation of the anatomical nasal cavity as well as the functional state of the sino nasal mucosa. This study investigates the role of DNE and its correlation with CT in the diagnosis and assessment of the severity of CRS.

The distribution of age and gender among CRS patients varies across studies, with no specific characteristics consistently identified for this condition. In our study, the average age was 51.57 ± 15.58 (mean ± SD) years, with the range of patients from 22 to 83 years. This corresponds with other research by Uwaneme SC et al was 43.4 ± 15.6 years, the age ranged from 18 to 83 years. In contrast, Ramadhin et al reported an average age of 37 years, with a range of 18 to 75 years.^[4,6]^ The majority of patients were male, comprising 67.1% (35/52 cases), the rate was lower in females at 32.69% (17/52 cases), and the male: female ratio was 2:1. A similar male preponderance was also found in the study of Ramadhin AK with the male of 61.5% and female of 38.5%^[4]^ and the proportion by Krishniya P was 66% and 34%, respectively.^[12]^

Nasal discharge (96.2%), nasal congestion (86.5%), and sneezing (67.3%) were identified as the most common presenting clinical features in this study, whereas other symptoms such as hyposmia/anosmia (3.9%) and facial pain/pressure (19.2%) were observed at lower frequencies. Our findings are consistent with those of other studies. For instance, Uwaneme SC et al^[6]^ observed nasal discharge in 95.0% of cases, nasal congestion in 92.5%, and sneezing in 47.5%. Similarly, Ramadhin AK et al reported high percentages of nasal discharge and nasal congestion, at 85.9% and 59%, respectively, but noted that headache was the most common symptom, affecting 94.4% of patients.^[4]^

Using DNE, the otorhinolaryngologist can accurately identify changes in nasal mucosal, nasal cavity deformities, nasal polyps, and secretions. Our results in Table 3 showed that bilateral nasal mucosal edema in the middle meatus was a common finding on endoscopy. This is corroborated by studies conducted by Uwaneme SC et al, who reported findings of middle nasal passage fluid in 56.7% of cases and nasal polyps in 32.55%.^[6]^ Additionally, Dawood MR et al, in their study examining the correlation between CT and endoscopy in 80 patients with CRS, found that mucosal edema was present in 63.75% of cases, with bilateral mucosal edema accounting for 88.24% and unilateral mucosal edema for 11.76%. Nasal polyps were observed in 22.5% of cases, with bilateral polyps accounting for 88.8% and unilateral polyps for 11.2%. Nasal fluid was present in 73.5% of cases, with unilateral fluid in 13.6% and bilateral fluid in 86.4%.^[13]^ The average Lund-Kennedy score was found to be 3.83 ± 1.37, indicating its utility in the diagnosis of CRS. Our result is consistent with Abhishek KR, who reported an average Lund-Kennedy score of 4.1 ± 3.1 in a study of 78 CRS patients^[4]^ and higher compared to that reported by Aslan F. in 2017, which was 2.4 ± 1.1.^[14]^

In imaging, CT is a helpful technique for evaluating CRS because it effectively assesses anatomical abnormalities and provides a detailed anatomic map for surgical planning. In the present study, the pathology in the maxillary sinus was the most commonly involved at 61.6% followed by the ethmoidal sinus at 57.7%, sphenoid sinus at 15.4%, whereas the frontal sinus was the least at 7.7% other studies.^[15,16]^ Uwaneme SC et al reported lesions in the maxillary sinus at 67.5% and ethmoid sinus at 50.8%; however, those in the sphenoid sinus and frontal sinus were more prevalent than in our study at 20.3% and 30.8%, respectively.^[6]^ In 2020, Sriprakash V et al reported lesions in the maxillary sinus accounted for 88%, with 38% being bilateral. Additionally, lesions in the ethmoid sinus constituted 54% (22% bilateral), while those in the sphenoid and frontal sinuses were 24% and 32%, respectively.^[4]^ Dawood MR’s study also reported similar findings.^[13]^ The location and severity of sinus lesions and ostiomeatal obstruction are evaluated according to the Lund-Mackay System with an average score of 4.3 ± 2.4. The scores of 3 to 4 need to be considered clinical and endoscopic evidence of the disease, whereas 5 or greater was considered diagnostically “positive” confirming the presence of disease. Ramadhin AK et al studied the correlation between endoscopy and CT scans had the average Lund-Mackay score was higher at 8.35 ± 6.03.^[4]^

In our study, the DNE correlated well with the CT scan findings. Positive findings on both endoscopy and CT scans were observed in 44 patients, while 2 cases with positive CT scan results were not detected by endoscopy among the 46 patients. Of the 6 patients with negative endoscopic findings, 5 also had negative CT scan results. Notably, 1 patient exhibited positive findings on endoscopy despite negative CT scans. DNE was more effective in identifying small polyps in the middle meatus, which may not be visible on CT scans. Research indicates that polyps are more frequently detected by nasal endoscopy than by CT.^[17,18]^ In addition, polyps are known to have nonspecific features on coronal CT scans compared to nasal endoscopy evaluation.^[19]^

Using endoscopy to corroborate findings, compared to CT scans was found to be 95.65%, with a specificity of 83.33%, PPV was 97.78%, and NPV of 71.43% were recorded. The high PPV, specificity, and sensitivity revealed that accurate diagnosis of CRS can be based on nasal endoscopic findings of mucosal edema, the presence of secretion, and/or polyps following the American academy of otolaryngology—head and neck surgery guidelines. However, the NPV of 71.43% means that negative findings on nasal endoscopy may not accurately rule out CRS in the patients. In a 2018 study, Ramadhin et al reported a correlation between DNE and CT in 78 patients of CRS, the sensitivity, specificity, PPV, and NPV were calculated as 92.7%, 91.3%, 96.2%, and 84% respectively, confirming that the addition of nasal endoscopy improves diagnostic accuracy for CRS.^[4]^ Subsequently, Sriprakash V et al evaluated the use of DNE and CT as effective tools for the detection and diagnosis of CRS. The sensitivity of DNE over CT was 95.6% while the specificity was 80%. The PPV and the NPV were 97.7% and 66.7%, respectively.^[5]^ In 2020, Uwaneme SC et al noted DNE is equally helpful and sometimes complementary to CT scans in the diagnosis of CRS with the sensitivity, specificity, PPV, and NPV of nasal endoscopy being 73.3%, 85.3%, 92.7%, and 55.8%, respectively.^[6]^ Recently, the findings of Singh AK et al in 2025 found that diagnostic nasal endoscopy demonstrated a sensitivity of 90.91% and a specificity of 84.61%, with a PPV of 93.48% and an NPV of 78.57%, demonstrating the role of DNE as a diagnostic tool in CRS and supporting its complementary use with CT for optimal CRS management.^[20]^

The observed slight variability in sensitivity and specificity among studies can be explained by several factors. Variability in patient populations and disease severity strongly influences results, with a higher proportion of patients with advanced CRS and CRS with nasal polyps (CRSwNP) tending to report higher sensitivity, while those with milder CRS or CRS without nasal polyps (CRSsNP), report lower sensitivity and NPV. Differences in diagnostic criteria also play an important role; for example, some authors classified minimal mucosal thickening on CT or mild edema on endoscopy as abnormal, increasing sensitivity but reducing specificity, while others required more advanced findings such as complete opacification or visible polyps, improving specificity but lowering sensitivity. In addition, factors related to imaging diagnostic standards, endoscopic equipment, study design, and sample size may contribute to heterogeneity across reports. Despite these sources of variability, the overall evidence consistently indicates and reinforces the high diagnostic value of DNE and its complementary role to CT in the evaluation of CRS.

On the other hand, CRS comprises distinct phenotypic subtypes, including CRSwNP and CRSsNP, which may affect diagnostic accuracy and outcomes. Endoscopy is typically more sensitive in CRSwNP due to visible polypoid changes, while CRSsNP, particularly localized forms, may present with minimal or absent endoscopic signs despite abnormal CT findings. In our study, we observed a strong concordance between endoscopic and CT findings. Thus, DNE may serve as an effective first-line diagnostic tool to support clinical suspicion or reduce the need for immediate CT imaging in situations where CT is not readily accessible. CT scans should be reserved for cases with negative or inconclusive endoscopic results but persistent suspicion of CRS, or in patients being considered for surgical intervention after failed medical therapy. This approach may help improve diagnostic efficiency while minimizing unnecessary radiation exposure and reducing financial burden, particularly in resource-limited healthcare settings.

The present study has several limitations. The sample size was relatively small, and the number of patients with visible nasal polyps was limited. Therefore, we were unable to stratify patients into CRSwNP and CRSsNP subgroups for meaningful statistical analysis. Additionally, in several cases, polyps were small or not consistently visible across both imaging and endoscopic assessment, limiting phenotypic classification reliability. Finally, the diagnostic findings from nasal endoscopy and CT scans were not compared with detailed parameters. Future studies with larger sample sizes need to evaluate the diagnostic utility of endoscopy within specific CRS subtypes in clinical application. This may help identify specific clinical scenarios where DNE offers clear advantages or requires complementary imaging for optimal diagnosis.

## 5. Conclusion

This study investigated the correlation between nasal endoscopy and CT scans, demonstrating that DNE is a valuable diagnostic test with an efficacy comparable to that of CT in diagnosing CRS. Additionally, CT imaging offers detailed insights into anatomical variations and sinus pathologies, which are essential for surgical planning and evaluating treatment outcomes. Therefore, both DNE and CT scans serve as useful diagnostic tools in the management of CRS and complement each other in achieving a more accurate diagnosis. Our findings reveal the practical value of DNE in primary diagnostic evaluation, particularly in settings where CT access is limited. Future research should focus on stratifying CRS patients by phenotype to determine how endoscopy performs within each subgroup.

## Acknowledgments

We express our sincere appreciation to all members of the Department of Otolaryngology, Head and Neck Surgery, 103 Military Hospital for their assistance and cooperation.

## Author contributions

**Conceptualization:** Tram Anh Do.

**Data curation:** Thanh Nam Quan.

**Formal analysis:** Thanh Nam Quan.

**Investigation:** Thanh Nam Quan, Tram Anh Do.

**Methodology:** Thanh Nam Quan, Tram Anh Do.

**Project administration:** Duc Thuan Nghiem.

**Software:** Tram Anh Do.

**Supervision:** Duc Thuan Nghiem.

**Validation:** Duc Thuan Nghiem.

**Visualization:** Duc Thuan Nghiem.

**Writing – original draft:** Tram Anh Do.

**Writing – review & editing:** Tram Anh Do.
